# Patients with uninjured lungs may also benefit from lung-protective ventilator settings

**DOI:** 10.12688/f1000research.12225.1

**Published:** 2017-11-22

**Authors:** Roger Alencar, Vittorio D'Angelo, Rachel Carmona, Marcus J Schultz, Ary Serpa Neto

**Affiliations:** 1 Department of Critical Care Medicine, Hospital Israelita Albert Einstein, São Paulo, Brazil; 2 School of Medicine, Hospital Israelita Albert Einstein, São Paulo, Brazil; 3 Deptartment of Intensive Care, Academic Medical Center, Amsterdam, Netherlands; 4 Laboratory of Experimental Intensive Care and Anesthesiology (L·E·I·C·A), Academic Medical Center, Amsterdam, Netherlands; 5 Mahidol Oxford Tropical Medicine Research Unit (MORU), Mahidol University, Bangkok , Thailand

**Keywords:** lung-protective ventilator, ventilator–induced lung injury, acute respiratory distress syndrome

## Abstract

Although mechanical ventilation is a life-saving strategy in critically ill patients and an indispensable tool in patients under general anesthesia for surgery, it also acts as a double-edged sword. Indeed, ventilation is increasingly recognized as a potentially dangerous intrusion that has the potential to harm lungs, in a condition known as ‘ventilator-induced lung injury’ (VILI). So-called ‘lung-protective’ ventilator settings aiming at prevention of VILI have been shown to improve outcomes in patients with acute respiratory distress syndrome (ARDS), and, over the last few years, there has been increasing interest in possible benefit of lung-protective ventilation in patients under ventilation for reasons other than ARDS. Patients without ARDS could benefit from tidal volume reduction during mechanical ventilation. However, it is uncertain whether higher levels of positive end-expiratory pressure could benefit these patients as well. Finally, recent evidence suggests that patients without ARDS should receive low driving pressures during ventilation.

## Introduction

Although mechanical ventilation is a life-saving strategy in critically ill patients and an indispensable tool in patients under general anesthesia for surgery, it also acts as a double-edged sword ^1^. Indeed, ventilation is increasingly recognized as a potentially dangerous intrusion that has the potential to harm lungs. Though frequently referred to as ‘ventilator-induced lung injury’ (VILI), this side effect may better be called ‘ventilation-induced lung injury’ as it is not the ventilator per se that causes harm but the way the ventilator is set ^1– 3^.

So-called ‘lung-protective’ ventilator settings aiming at prevention of VILI have been shown to improve outcomes in patients with acute respiratory distress syndrome (ARDS) ^4^. Nevertheless, ARDS remains a condition with high mortality and morbidity ^5^, and in light of this there has been a paradigm shift from treating to preventing ARDS. Over the last few years, there has been increasing interest in a possible benefit of lung-protective ventilation in patients under ventilation for reasons other than ARDS, such as critically ill patients ^1^ and surgery patients ^2, 3^.

After a brief summary of the existing evidence of benefit of lung-protective ventilation in patients with ARDS, this review discusses the potential role of these ventilation strategies in patients with uninjured lungs. We cite the literature according to the available ‘best’ evidence, where results of meta-analysis using individual patient data are seen as better evidence than conventional meta-analysis, followed by randomized controlled trials (RCTs) and finally observational studies. Where possible, we also cite the literature on ongoing RCTs.

## Lung-protective ventilation in patients with acute respiratory distress syndrome

In patients with ARDS, outcomes could be improved by adjusting three more or less simple ventilator settings: tidal volume, positive end-expiratory pressure (PEEP), and driving pressure ^6^. One way to protect the lungs of patients with ARDS is to prevent ‘volutrauma’ by using low tidal volumes ^7^. Pivotal RCTs performed almost 20 year ago showed that ventilation with low tidal volumes improved the survival of patients with ARDS ^4, 8^, a finding that was convincingly confirmed in one meta-analysis ^7^. The results of more recent investigations even suggest that a further reduction in tidal volume size, supported by the use of extracorporeal removal of carbon dioxide, could improve the survival of patients with severe forms of ARDS even further ^9, 10^.

Another way to protect the lungs of patients with ARDS is to prevent ‘atelectrauma’ by using higher PEEP ^11^. Three pivotal RCTs showed no benefit of higher PEEP ^12– 14^, but the results of one meta-analysis using individual patient data from these three RCTs compellingly suggested that patients with moderate or severe ARDS die less frequently when higher PEEP is used ^15^. Of note, the same meta-analysis also showed an association between use of high PEEP and a worse outcome in patients with mild ARDS. Nevertheless, a recent RCT showed that, in patients with moderate to severe ARDS, the use of lung recruitment maneuvers and titrated higher levels of PEEP were associated with higher mortality, increased risk of barotrauma, and longer duration of ventilation compared with lower levels of PEEP ^16^. Indeed, these findings do not support the routine use of lung recruitment maneuver and higher PEEP in these patients.

Finally, the lungs of patients with ARDS could be protected against so-called ‘energytrauma’ by using low driving pressures ^17^. Indeed, one large meta-analysis using individual patient data from nine RCTs strongly suggests that patients with ARDS have a better outcome when driving pressures remain low ^18^ and these findings were confirmed in a recent study in a large population of patients with ARDS ^19^. Of note, none of the original RCTs used in this meta-analysis tested directly whether a low driving pressure reduced mortality; we thus need to be careful in interpreting the findings. Nevertheless, the results of yet another meta-analysis using individual patient data also suggest that outcomes improve when driving pressures are low, this time in ARDS patients receiving extracorporeal membrane oxygenation because of refractory hypoxemia ^20^.

## Evidence of benefit of lung-protective ventilation strategies in patients with uninjured lungs

For a long time, prevention of VILI was considered relevant only in patients with ARDS and only when mechanical ventilation was applied for a substantial period of time (that is, for days) ^21, 22^. Recent investigations, however, show that lung injury can develop in all types of patients, thus also in patients with uninjured lungs, and also when ventilation is applied for short periods of time (that is, for hours) ^23, 24^. Several types of patients thus could benefit from lung-protective ventilation strategies ^25^.

### Tidal volume

Until recently, ventilation strategies with high tidal volumes were preferred over strategies with low tidal volumes in patients with uninjured lungs, as ventilation with high tidal volumes could prevent or at least reduce the amount of atelectasis ^26^, thereby preventing the need for high oxygen fractions. However, two individual patient data meta-analyses strongly suggest that intensive care unit (ICU) patients with uninjured lungs could also benefit from ventilation with low tidal volumes ^27, 28^. Paradoxically, in contrast with the results of these two meta-analyses, the recent ‘Practice of Ventilation in critically ill patients without ARDS’ (PRoVENT) study, an international prospective study of ventilation practices in ICU patients without ARDS, found no association between tidal volume size and diverse outcomes ^29^. However, in the PRoVENT study, tidal volumes were noticeably lower in comparison with almost all preceding investigations, and also the range of tidal volumes was much smaller ^29^. Secondly, results from a meta-analysis using individual patient data need to be looked at with caution, as such analyses sometimes are little more than so-called ‘per protocol’ analyses in which patients who actually received the intervention of interest are compared with patients who did not receive that intervention ^30^. Intentional as well as unintentional reasons could be responsible for not receiving the intervention of interest, in this case low tidal volumes, and some of these reasons, recognized or unrecognized, could have an association with the outcome ^30^. For example, in ICU patients with severe acidosis, who often need ventilation with high tidal volumes to have an acceptable arterial pH, (reasons for) severe acidosis could have a much stronger association with outcome than tidal volume size. One recent study in France showed that implementation of the use of low tidal volumes is not so simple, and many factors such as the use of spontaneous modes of ventilation, higher metabolic demands, and lower sedation levels could be responsible for it ^30, 31^. Nevertheless, a recent study in the US suggested that tidal volume reduction after out-of-hospital cardiac arrest could be associated with favorable neurocognitive outcome, more ventilator-free days, and even more shock-free days ^32^. Altogether, these data suggest that, in fact, low tidal volume is physiological tidal volume, as suggested by animal studies ^33^.

Whether ICU patients with uninjured lungs truly benefit from a reduction in tidal volume size thus remains uncertain. Two ongoing RCTs could answer the question of whether tidal volumes should be kept low in ICU patients with uninjured lungs. The ‘Protective Ventilation in patients without ARDS at start of ventilation’ (PReVENT) trial ^34^ is a Dutch national multicenter RCT that compares ventilation with a tidal volume between 4 and 6 mL/kg predicted body weight (PBW) with ventilation with a tidal volume between 8 and 10 mL/kg PBW in 952 invasively ventilated ICU patients without ARDS. The ‘Preventive Strategies in Acute Respiratory Distress Syndrome’ (EPALI) trial ^35^ is a Spanish national multicenter RCT comparing ventilation with a tidal volume between 4 and 6 mL/kg PBW with ventilation with a tidal volume between 8 and 10 mL/kg PBW in 400 invasively ventilated ICU patients at risk for ARDS. The results of these two RCTs are expected soon.

Patients receiving ‘emergency’ ventilation could also benefit from use of low tidal volumes. A ventilation protocol including use of low tidal volumes in an emergency department (ED) was feasible and associated with improved outcomes not only in emergency patients with ARDS ^36^ but also in emergency patients with uninjured lungs ^37^. Yet implementation of lung-protective ventilation with low tidal volumes remains poor in these patients ^38^.

One individual patient data meta-analysis suggests that ventilation with low tidal volumes results in less postoperative pulmonary complications in surgery patients under general anesthesia ^39^. This, however, was not found in the recent ‘Local Assessment of Ventilatory Management During General Anesthesia for Surgery and effects on Postoperative Pulmonary Complications’ (LAS VEGAS) study; this international prospective study on ventilation practices in the operating room found no association between tidal volume size and diverse outcomes ^40^. However, in the LAS VEGAS study, as in the above-cited PRoVENT study, tidal volumes were also lower than almost all preceding studies, and the range of tidal volumes was remarkably small ^40^.

### Positive end-expiratory pressure

Ventilation with low tidal volumes could induce alveolar instability, resulting in cyclic opening and closing of alveoli ^1, 11^, frequently referred to as ‘tidal recruitment’. PEEP may keep these lung regions open at the end of expiration and thus prevent tidal recruitment. However, use of PEEP comes ‘at a price’, as PEEP could also cause regional overdistension, in particular of the non-dependent lung parts, and has negative effects on cardiac performance ^41^. The balance between benefit and harm of PEEP may very well differ between patients receiving mechanical ventilation for various reasons other than ARDS ^39, 42, 43^.

One conventional meta-analysis of studies in critically ill patients with uninjured lungs clearly showed that evidence of benefit of PEEP is lacking, when focusing on important patient-centered endpoints such as mortality and duration of ventilation ^43^. The PRoVENT study showed higher PEEP in ICU patients at risk for ARDS compared with patients not at risk for this complication ^29^, although the differences were small. A recent RCT in ICU patients after cardiac surgery showed that high PEEP resulted in less severe pulmonary complications ^44^. It should be noted, though, that the patients in this RCT probably had lung injury ^45^.

Whether ICU patients with uninjured lungs could benefit from higher levels of PEEP is currently uncertain. The ‘Restricted versus Liberal positive end-expiratory pressure in patients without Acute respiratory distress syndrome’ (RELAx) trial, a Dutch national multicenter RCT that compares ventilation with PEEP of 8 cm H _2_O against restricted PEEP (lowest possible) in 980 invasively ventilated ICU patients with uninjured lungs, may help to answer the question of what the best level of PEEP in these patients is ^46^.

An implementation study focusing on the liberal use of PEEP in ED patients resulted in higher PEEP in patients with ARDS ^36^ but also in patients with uninjured lungs ^37^. The latter was associated with improved outcomes. Of note, however, the investigators focused not only on implementation of liberal use of PEEP but also on use of low tidal volume, head-of-bed elevation, and timely oxygen weaning ^36, 37^, which all could explain the better outcomes.

The ‘Protective Ventilation using High versus Low positive end-expiratory pressure’ (PROVHILO) trial, an international RCT that compared high PEEP versus low PEEP during intraoperative ventilation in surgery patients, showed that high PEEP did not prevent postoperative pulmonary complications ^42^. Nevertheless, it is important to note that the PROVHILO trial did not assess moderate levels of PEEP, such as 5–8 cm H _2_O; thus, the effects of these levels of PEEP in surgical patients are still open for debate.

### Driving pressure

There has been only one single study that showed an association between driving pressure and development of ARDS in patients with uninjured lungs ^47^. This study showed a better outcome of brain injury patients who received ventilation with low driving pressures. Studies on driving pressure during ventilation in emergency patients are lacking at present.

Most evidence of benefit of ventilation with low driving pressures in patients with uninjured lungs comes from one individual patient data meta-analysis of studies in surgery patients receiving intraoperative ventilation ^48^. This analysis shows an independent association not only between absolute driving pressures and the occurrence of postoperative pulmonary complications but also between changes in driving pressure due to changes in PEEP and occurrence of postoperative pulmonary complications. Recruitment of lung tissue through use of higher PEEP could explain, in part, the decrease in driving pressure ^41, 45^. The LAS VEGAS study also showed an association between higher driving pressure and development of postoperative pulmonary complications ^40^.

The ‘Individualized Perioperative Open Lung Ventilatory Strategy’ (iPROVE) study will assess whether an individualized strategy of ventilation combining recruitment maneuvers and PEEP titration according to the compliance of the respiratory system is beneficial in surgical patients at risk for postoperative pulmonary complications ^49^. Also, the ‘Driving Pressure during General Anesthesia for Abdominal surgery’ ** (DESIGNATION) study will test whether a PEEP titration aiming at the lowest driving pressure possible during surgery, compared with a standard PEEP of 5 cm H _2_O, decreases the incidence of postoperative pulmonary complications in patients at risk for postoperative pulmonary complications and undergoing abdominal surgery.

## Conclusions

The lungs of patients with uninjured lungs may very well be as vulnerable to the harmful effects of mechanical ventilation as the lungs of patients with ARDS, and probably the same three ventilator settings—tidal volume, PEEP, and driving pressure—play a role. The results of ongoing RCTs are eagerly awaited.
